# Carbonate Production by Benthic Communities on Shallow Coralgal Reefs of Abrolhos Bank, Brazil

**DOI:** 10.1371/journal.pone.0154417

**Published:** 2016-04-27

**Authors:** Vanessa Moura dos Reis, Cláudia Santiago Karez, Rodrigo Mariath, Fernando Coreixas de Moraes, Rodrigo Tomazetto de Carvalho, Poliana Silva Brasileiro, Ricardo da Gama Bahia, Tito Monteiro da Cruz Lotufo, Laís Vieira Ramalho, Rodrigo Leão de Moura, Ronaldo Bastos Francini-Filho, Guilherme Henrique Pereira-Filho, Fabiano Lopes Thompson, Alex Cardoso Bastos, Leonardo Tavares Salgado, Gilberto Menezes Amado-Filho

**Affiliations:** 1 Instituto de Pesquisas Jardim Botânico do Rio de Janeiro, Rio de Janeiro, Rio de Janeiro, Brazil; 2 Instituto Oceanográfico, Universidade de São Paulo, São Paulo, São Paulo, Brazil; 3 Departamento de Invertebrados, Museu Nacional do Rio de Janeiro, Rio de Janeiro, Rio de Janeiro, Brazil; 4 Departamento de Biologia Marinha, Universidade Federal do Rio de Janeiro, Rio de Janeiro, Rio de Janeiro, Brazil; 5 Departamento de Engenharia e Meio Ambiente, Universidade Federal da Paraíba, Rio Tinto, Paraíba, Brazil; 6 Instituto do Mar, Universidade Federal de São Paulo, Santos, São Paulo, Brazil; 7 Departamento de Oceanografia, Universidade Federal do Espírito Santo, Vitória, Espírito Santo, Brazil; Università di Genova, ITALY

## Abstract

The abundance of reef builders, non-builders and the calcium carbonate produced by communities established in Calcification Accretion Units (CAUs) were determined in three Abrolhos Bank shallow reefs during the period from 2012 to 2014. In addition, the seawater temperature, the irradiance, and the amount and composition of the sediments were determined. The inner and outer reef arcs were compared. CAUs located on the inner reef shelf were under the influence of terrigenous sediments. On the outer reefs, the sediments were composed primarily of marine biogenic carbonates. The mean carbonate production in shallow reefs of Abrolhos was 579 ± 98 g m^-2^ y^-1^. The builder community was dominated by crustose coralline algae, while the non-builder community was dominated by turf. A marine heat wave was detected during the summer of 2013–2014, and the number of consecutive days with a temperature above or below the summer mean was positively correlated with the turf cover increase. The mean carbonate production of the shallow reefs of Abrolhos Bank was greater than the estimated carbonate production measured for artificial structures on several other shallow reefs of the world. The calcimass was higher than the non-calcareous mass, suggesting that the Abrolhos reefs are still in a positive carbonate production balance. Given that marine heat waves produce an increase of turf cover on the shallow reefs of the Abrolhos, a decrease in the cover represented by reef builders and shifting carbonate production are expected in the near future.

## Introduction

Coral reefs are in decline on a global scale [1–4] and several factors related to this decline stem from anthropogenic activities [5]. The main threats to coral reef ecosystems are overfishing, contamination from land-based activities, marine pollution, waste water release in coastal areas and increased sedimentation [6,7]. These activities can degrade coral reefs on a local scale, making them less resilient to climate change [8]. Ocean warming and acidification can result in coral mortality and reduce the calcification rates of reef-building corals [9,10]. Sedimentation and eutrophication can reduce coral recruitment, modify trophic structure, and, as a consequence, alter biodiversity [11].

Corals and crustose coralline algae are considered the main reef builders organisms in Abrolhos reefs as in other Brazilian reefs [12,13]. Reported impacts include increased coral diseases in 2005 [14,15] and reduced populations of herbivorous reef fishes [16,17], which triggered indirect effects on the trophic chain and affected reef system resilience [18]. The first bleaching event at Abrolhos reefs was recorded during the summer of 1993–1994, when 50 to 90% of the coral colonies were bleached after the occurrence of a marine heat wave associated with an El Niño global episode [19]. In the following decade, several successive bleaching events occurred [20].

Coral reefs are, in general, quite sensitive to sea temperature anomalies [21]. Bleaching processes and coral infectious diseases have been increased by seawater temperature rise [22]. In calcareous algae, higher seawater temperatures can cause a decrease in calcification rates or an increase in mortality [23,24]. In addition, elevated temperature can contribute to phase shifts in coral reefs from the original dominance of reef-building calcified organisms to a preponderance of fleshy seaweed [25] or turf [26] with negative impacts to other species The structural complexity of habitats afforded by the reef-builders, provides shelter and protection to benthic and pelagic organisms.

A long-term monitoring program at the Abrolhos reefs showed an increase in turf algal cover from 2006 to 2008 [26]. The current definition of ‘turf’ is unlikely to refer to a single type of alga, but represents several types of micro and macroalgae which share an extensive low-lying morphology [27]. These benthic organisms were the most abundant (56% of living cover), indicating a phase shift in the Abrolhos reef ecosystem. Turfs can increase coral stress [28] and cause many deleterious effects, including tissue damage [29,30], fertility reduction [31] and coral recruitment failure [32,8].

Several previous field experiments have investigated the impact of abiotic factors in the colonization of encrusting communities in artificial substrates in coral reefs [33–36] and their carbonate production in the Caribbean [37–40], in the Central Pacific [41] and in the South Pacific [42], but not in the South Atlantic.

The evaluation of coral reef ecosystems can be performed based on investigations of the balance between the abundance of carbonate reef-builders and non-builders. This is one of the mostly widely used metric to evaluate reef condition, with the dominance of the reef-builders indicating a healthy ecosystem [26, 43]. Thus, a shift in community structure to a dominant position for non-builders, such as macroalgae and other groups, will lead to the loss of habitat complexity and biodiversity [22,44,45].

Recently, the assessment of colonization and carbonate production by benthic organisms was analyzed by using artificial structures named Calcification Accretion Units (CAUs) [41]. Monitoring benthic communities in different reef ecosystems simultaneously with environmental variables such as temperature, light intensity and associated sediments will allow the evaluation of the contribution of the reef-building community and the influence of these variables on the structure of the Abrolhos Bank reefs. Therefore, aim of this work is to determine the abundance of reef builders and non-builders in advanced stages of colonization and the carbonate production of communities established on artificial structures on the shallow reefs of Abrolhos Bank.

## Materials and Methods

### Study Sites

**Fig 1:**
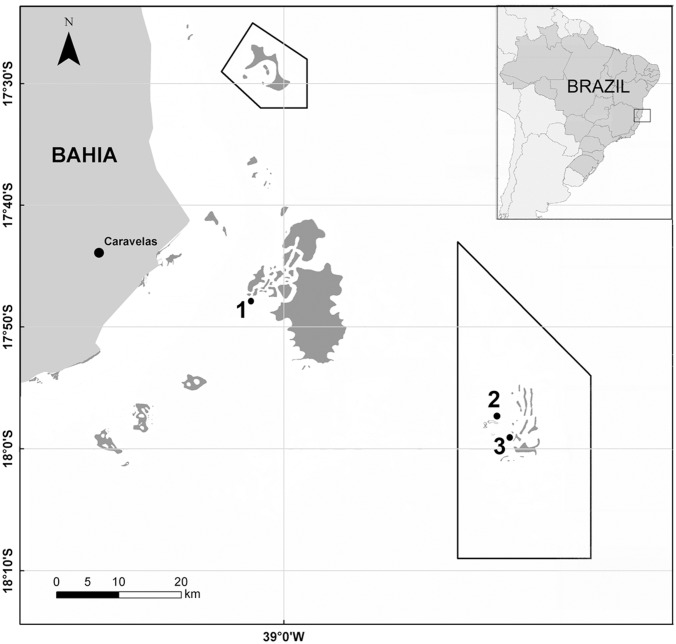
Map of Abrolhos Bank, eastern Brazil, showing study sites and marine protected areas. 1) Pedra de Leste, 2) Abrolhos Archipelago, 3) Parcel dos Abrolhos. Abrolhos Marine National Park is represented by polygons.

This study was carried out on reef tops of Pedra de Leste (PL)—inner shelf reef, and Abrolhos Archipelago (AA) and Parcel dos Abrolhos (PA)—outer shelf reefs (Fig 1).

The Pedra de Leste reefs (17°47′00.199” S 39°03′05.099” W) are the near-coast study site, approximately 10–15 km away from the shore, located in the Parcel das Paredes. They consist of shallow large reef banks, which are formed by pinnacles fused at their tops, and isolated pinnacles, surrounding the fused structures [13,46]. At this site, the experiment was conducted at a depth of approximately 3 m.

Abrolhos Archipelago (17°57′37.81” S 38°41′58.90” W) is located approximately 70 km east of the coast and is surrounded by fringing reefs that have developed over the rocky shore substrate [13]. At this site, the experiment was conducted at a depth of approximately 5 m.

The Parcel dos Abrolhos reefs (17°58′54.3” S 38°40′22.9” W) are located approximately 75 km east of the coast. They consist of isolated giant coral pinnacles, ¨*chapeirões*¨ which do not fuse to form bank reefs as they do in the coastal zone [13,47]. At this site, the experiment was conducted at a depth of approximately 8 m.

Only the outer reefs, AA and PA, are located within a no-take marine protected area, which was given the designation of Abrolhos Marine National Park in 1983. Research permits in AA and PA were provided by Abrolhos Marine National Park/Instituto Chico Mendes de Conservação da Biodiversidade (ICMBio).

### Experimental Design

**Fig 2:**
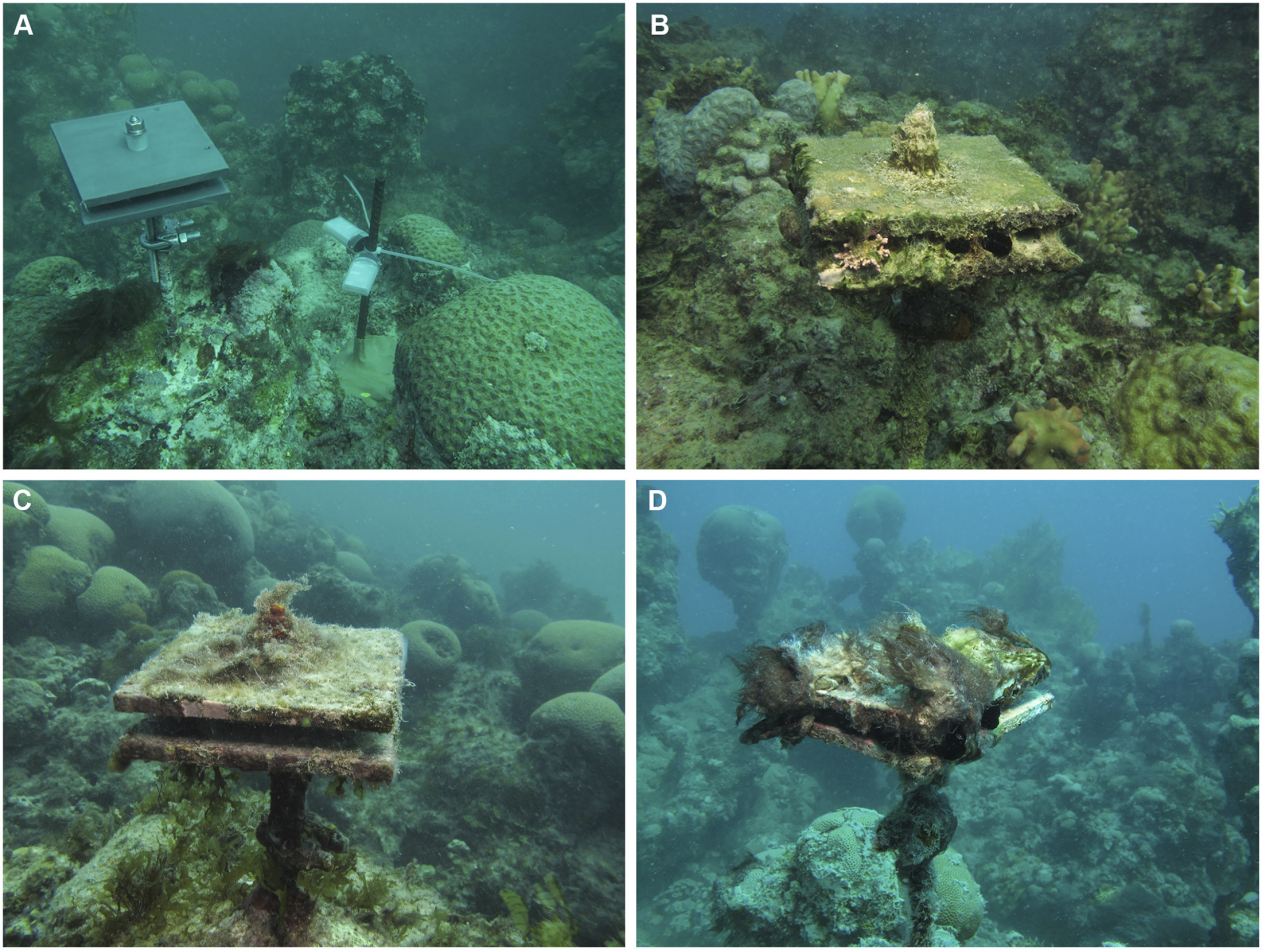
CAUs (Calcification Accretion Units) and HOBO sensors (light and temperature) in situ. A: CAU and HOBO sensors immediately after installation in the summer of 2013; B-D: CAUs in the summer of 2014 (after 1 year of colonization) at P. Leste, Abrolhos Archipelago and P. Abrolhos, respectively.

Artificial structures termed Calcification Accretion Units (CAUs) were used for the colonization assays (N = 6 per site, per year) (Fig 2) [41]. Each structure consisted of two horizontally superposed and spaced plates (10 cm × 10 cm each plate) of nontoxic PVC Type I (InterState Plastic Co., Sacramento, CA, USA). The total area of each CAU including the upper and lower surfaces of both plates was 400 cm^2^. CAUs have been tested in other reef-builder colonization studies in a different reef region [41] and have been shown to reproduce the structural complexity of a natural reef. CAUs were carefully deployed at approximately 20 cm above the reef tops.

The CAU temporal replicates were removed after one year in PL, AA and PA in two consecutive periods of the experiment: 2012–2013 and 2013–2014. In AA, other CAUs were also deployed for two years in the field during 2012–2014. Each CAU was carefully recovered after being wrapped in plastic bags to maintain the trapped sediment as well as the integrity of delicate organisms such as tunicates, bryozoans and turf-forming algae.

Light intensity and temperature sensors (HOBO Pendant^®^ Temperature/Light Data Logger 64K—UA-002-64 HOBOc) were deployed adjacent to the CAUs for one year in the field at the same time as the artificial structures.

After recovery, the CAUs were dismantled on plastic trays, and both sides (upper and lower surfaces) of the 2 PVC plates were photographed at high resolution (Nikon D3100 camera). The images were managed using Lightroom 5 software.

Plates were also preserved in 10% formalin in seawater and protected from light. The abundance of encrusting communities was determined based on the percentage area of CAUs’ plates covered by each taxa, estimated using the image analysis software ImageJ (NIH, Bethesda, Maryland, USA). The percentage of the area that was covered by organisms (percent cover) was determined by calculating the ratio between the area covered by organisms and the total area of the surface analyzed.

### Temperature and Light Intensity Measurements

The sensors were set to record light intensity and temperature at 1 h intervals over 1 year.

At all studied sites, monthly mean sea-surface temperatures were calculated between March of 2013 and February of 2014. At AA, daily mean temperatures were obtained for the summer during two consecutive years: 2013 and 2014. The summer mean temperatures were calculated for each year. The number of consecutive days with temperatures above or below the summer mean temperature was counted.

At all sampling sites, light intensity was measured (in LUX) for the first 10 days of sensors in the field during February of 2013. Photosynthetically active radiation (PAR) values were obtained based on a conversion factor in which one PAR unit was equivalent to 20 LUX units.

### Sediment Analysis

For sediment analysis, both plates of CAUs were previously rinsed with seawater in recipients. After 8 hours for sediments settled down in the recipients, a peristaltic pump was used to remove the seawater excess. Then sediments were centrifuged, dried and weighed. To determine the mineralogy of the sediments on the CAUs, a powder X-ray diffraction technique was used. The samples were ground twice for 10 min and sieved through 25 μm mesh. Large particles were reground until they passed through the sieve. X-ray data were collected using a PANalytical X’Pert Pro Multipurpose Powder Diffractometer (Bragg-Brentano geometry, CuKα radiation, generator: 40 mA and 40 kV). An angular range of 5 to 90° 2θ was measured with a step size of 0.05° and a 60 s counting time per step. Samples were prepared in triplicate, and each replicate sample was measured three times. Phase identification was performed with Panalytical X’Pert Pro V3 software, which was used to determine the diffraction peaks. A search and match algorithm was used with organic and inorganic databases to identify the crystals found in the samples. The automated mineral identification results were confirmed or refuted / amended using a technical analysis of the diffraction peaks. Phase quantification and lattice parameter determination were performed (N = 5 interactions) with the Rietveld refinement software MAUD. The software was also used to determine the percentage of Mg substitution in the calcite crystal lattice in the sediments (adapted method described by Titschack et al. [48]) by using *a* and *c*, the *c*/*a* ratio, and cell volume as refinement parameters.

### CaCO_3_ Production Estimate

To quantify carbonate production by the encrusting communities, the protocol established by Price et al [41] was used, with modifications. The colonized plates were cleaned in seawater, dried at 60°C and weighed. They were then submerged in 5% HCl for 48 h or until all CaCO_3_ was dissolved. The remaining non-calcareous tissue was scraped and vacuum filtered using pre-weighed 15 μm cellulose filter papers and dried at 60°C until the material became stable. These samples were weighed, and the non-calcareous mass was determined by subtracting the weight of the cellulose filters from the total. Finally, the calcimass was determined by subtracting the weight of the non-calcareous tissue and of the plates from the total mass of the CAUs. The total calcareous mass was divided by the total surface of each plate (200 cm^2^ –accounting the upper and lower surfaces), and the carbonate production calculated for each CAU was presented as g m^−2^ y^−1^.

### Data Analysis

Differences in each variable: deposited sediment quantity, temperature, photosynthetically active radiation, percent cover of reef-builders and non-builders, carbonate production and non-calcareous mass were evaluated with a permutational multivariate ANOVA (PERMANOVA) that included one, two or three fixed factors. The analyzed factors were the site (PL, AA and PA), the year (2012–2013 and 2013–2014), the period of colonization (1 or 2 years) and the category of organisms (builders and non-builders). Multivariate and univariate analyses were based on Bray—Curtis dissimilarities (percent cover of reef organisms and carbonate production) and Euclidean distances (abiotic factors). P-values were calculated from 4999 unrestricted permutations of the raw data.

To evaluate whether variation in measured abiotic variables significantly contributed to explaining the variation in percent cover of main organisms among the sites, a distance-based redundancy analysis was tested. Multivariate multiple regression, using the DISTLM (Distance-based Linear Models) routine, was then used to test the significance of these relationships. Simple linear correlations were performed between the number of consecutive days with temperatures above and below the summer mean temperature and the percent cover of main organisms.

Statistical analyses (PERMANOVA, DISTLM) were conducted using PRIMER (version 6) + PERMANOVA software. Pairwise comparisons were used, when appropriate, to resolve differences among levels of significant factors (p < 0.05).

## Results

### Sea-Surface Temperature

**Fig 3:**
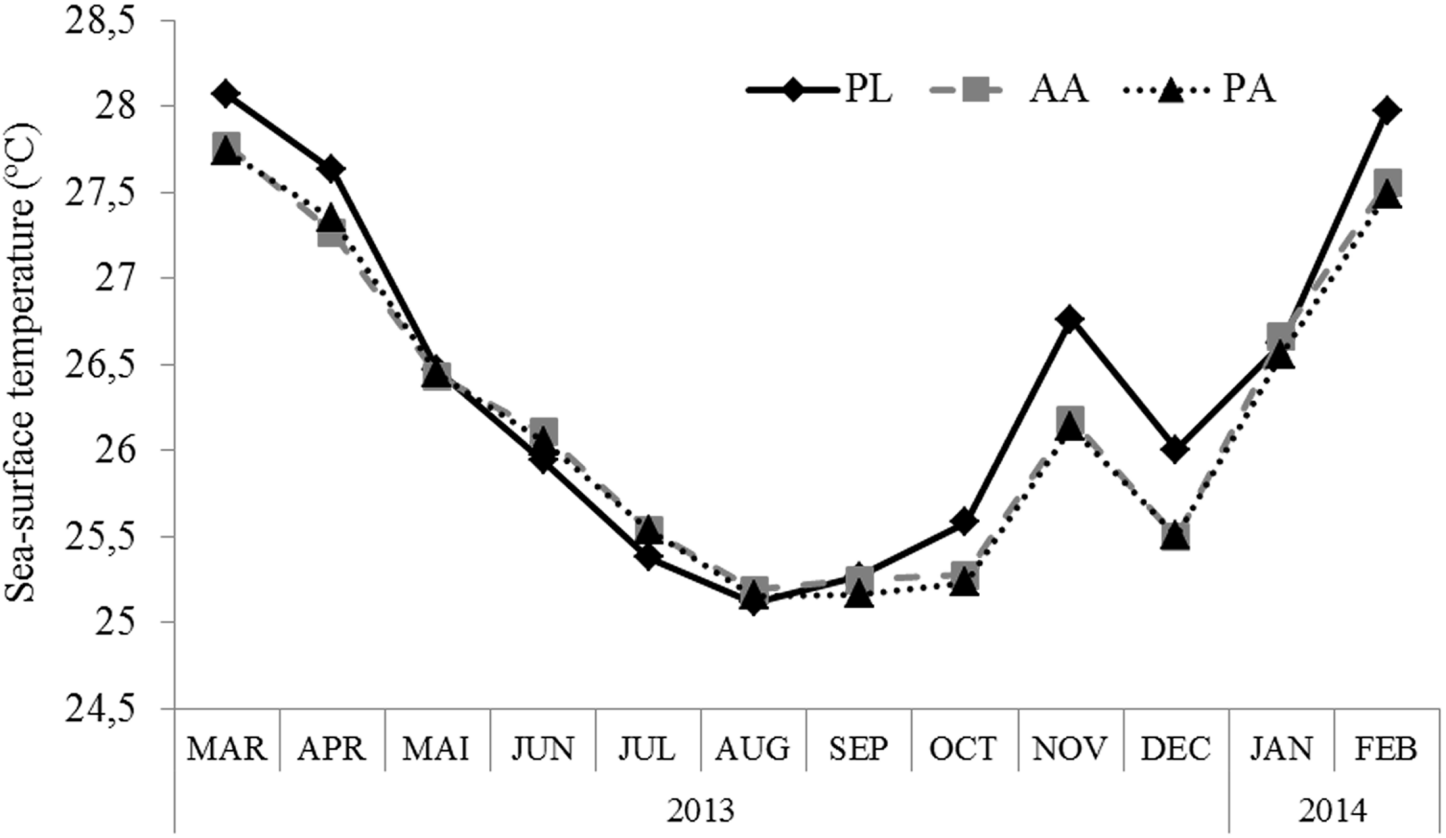
Monthly mean sea-surface temperature of shallow reefs from March 2013 to February 2014. PL—Pedra de Leste, AA—Abrolhos Archipelago, PA—Parcel dos Abrolhos.

Monthly sea-surface temperatures during 2013–2014 did not differ among the studied sites (PERMANOVA, F = 1.675, p = 0.1886). Independent of the site, the monthly mean seawater temperatures varied between 25°C and 28°C (Fig 3). The lower mean temperature values were measured at the end of autumn, winter and the beginning of spring; the higher values were measured at the end of spring, summer and the beginning of autumn.

**Fig 4:**
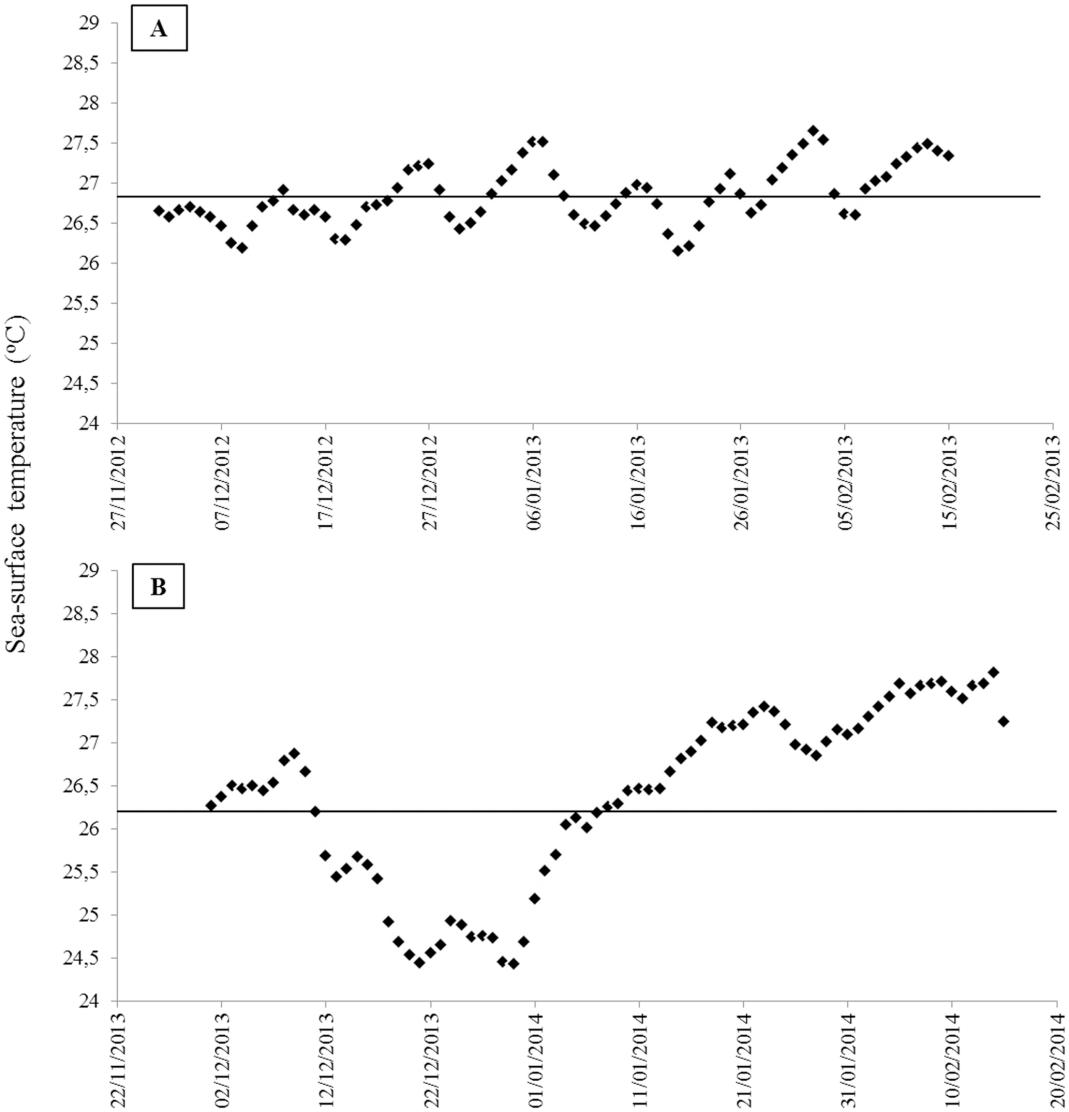
Daily mean sea-surface temperatures during December and January in Abrolhos Archipelago. A) 2012–2013, B) 2013–2014. Solid lines represent the mean summer temperature in both years.

The daily mean sea-surface temperatures differed between the summer of 2012–2013 and 2013–2014 (PERMANOVA, F = 3.283, p < 0.0001) (Fig 4).

During the summer of 2012–2013, the daily mean sea-surface temperature was 26.8°C. In this summer, three short events involving seawater temperature increases were recorded, with a maximum of six consecutive days on which temperatures were higher than the average of this period.

During the summer of 2013–2014, the daily mean sea-surface temperature was 26.3°C. During this summer, two events of seawater temperature rise were recorded, with 9 and 18 consecutive days with temperatures higher than the average of this period and one event of temperature decrease, with 14 consecutive days of mean temperatures below 26.3°C.

### Light Intensity

**Fig 5:**
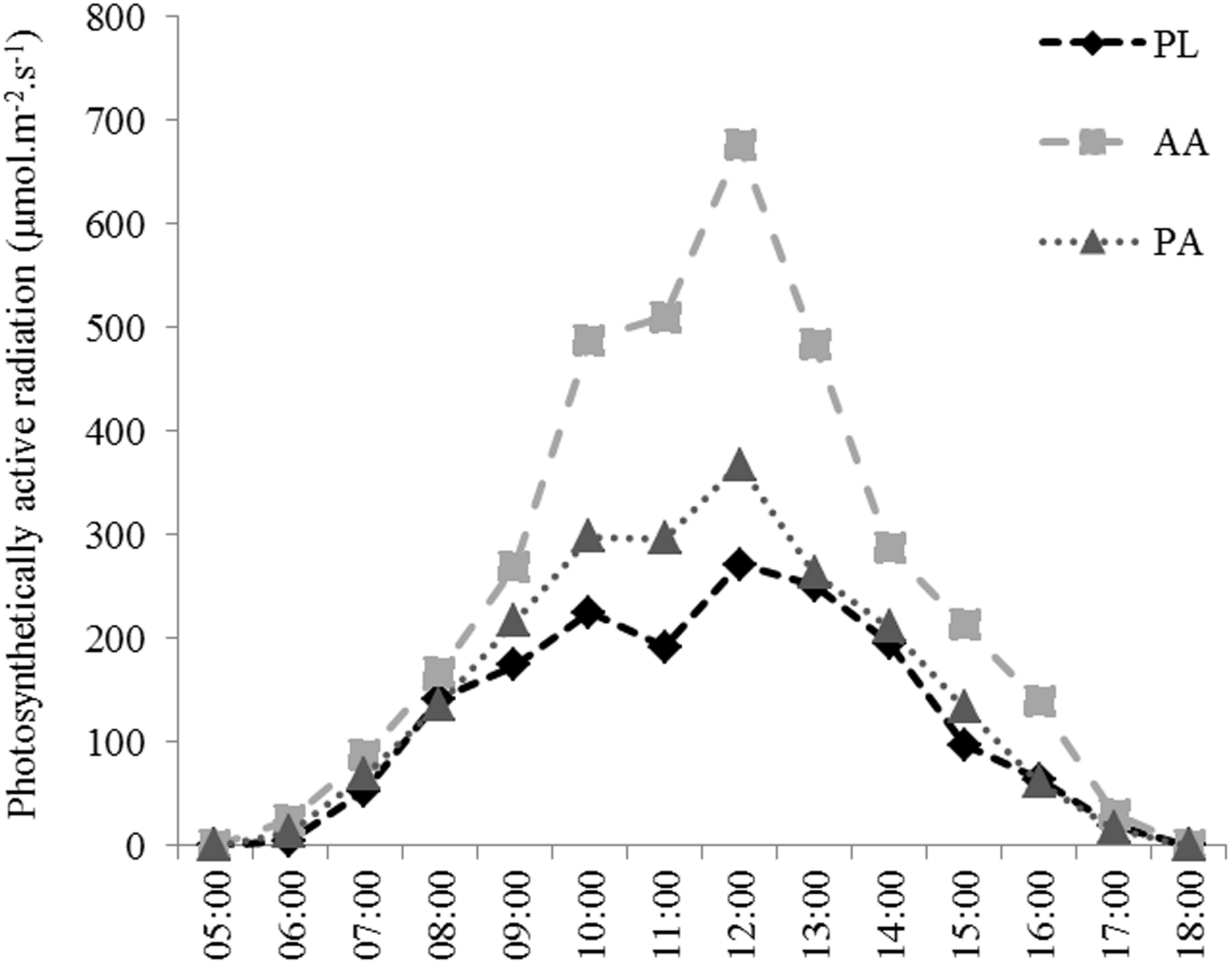
Mean light intensity measured during the day on the shallow reefs between March of 2013 and February 2014.

At all three sites, the daily cycle of photosynthetically active radiation (PAR) was obtained from observations covering the period 5:00 to 18:00 h, the daily light period, with the highest values measured at 12:00 h (Fig 5).

The photosynthetically active radiation (PAR) differed among the sites (PERMANOVA, F = 15.508, p = 0.0002; Fig 5). AA showed the highest values of irradiance between 10:00 to 16:00 h. However, no difference was observed between 5:00 to 9:00 h and after 17:00 h. The irradiances did not differ between PA and PL.

### Sediments on CAUs—Amount and Mineralogy

The mean amounts of deposited sediments (minerals and organic) on the CAUs at PL (1.17 ± 0.1 g) were higher than those measured at PA (0.6 ± 0.03 g) and AA (0.15 ± 0.01 g) (PERMANOVA, F = 11.628, p = 0.01).

Five mineral types were identified: calcite; high magnesium calcite; aragonite; kaolinite; and quartz (S1 Table). Calcite containing more than 4% wt of MgCO_3_ is conventionally defined as high Mg-calcite [49]. Mg-calcite and aragonite constituted more than 80% of minerals in sediments from all sites. Kaolinite and quartz (terrigenous sediments) were observed almost exclusively at PL, where they represented 18% of the sediment composition.

Based on the classification of terrigenous and marine sediments, the results showed that the CAUs located at PL, the inner shelf reef, showed an influence of terrigenous sediments composed of 18.3% kaolinite and quartz. In contrast, at the CAUs located at AA and PA, the outer reefs, these minerals were essentially not detected (0.4%), indicating that the sediments over the CAUs in those sites were mainly composed of marine biogenic calcium carbonates. The most abundant mineral in the CAU sediments was Mg calcite in all sites

The biogenic mineral composition at different locations showed the following characteristics: AA presented a higher percentage of Mg-calcite as well as a higher ratio of this mineral in relation to aragonite (Mg-calcite:aragonite); PA showed the highest amount of aragonite and PL the lowest amount; finally, the percentage of calcite was similar at PL and PA and lower at AA (S1 Table).

### Abundance of Colonizers

In total, 70 taxa were identified belonging to calcareous and non-calcareous encrusting organisms (S2 Table).

After one year of colonization, the highest coverage on CAU plates was observed in AA (62 ± 5%). PL showed 50 ± 6 percent cover, PA 48 ± 6 percent cover. The most abundant groups of organisms were crustose coralline algae (CCA), with more than 20% mean cover, followed by turfs and ascidians with more than 10%. Bryozoans and fleshy algae covered 5% and 2%, respectively. Corals, sponges, mollusks and polychaetes were less abundant, with up to 2% of mean cover. Foraminifers and zoanthids were rare, with less than 1% of mean cover (Table 1).

**Table 1:** Mean (± standard error) abundance (%) of colonizer organism groups for sites (PL, AA and PA), years (2012–2013, 2013–2014, 2012–2014) and periods of colonization (1 and 2 years) analyzed.

Groups	2012–2013			2013–2014			Mean (1 year)	2012–2014 (2 years)
	PL	AA	PA	PL	AA	PA		AA
Ascidians (NB)	8.6 ± 2.1	6.2 ± 3.1	13.1 ± 4.7	11.7 ± 3	10.7 ± 2	18.2 ± 1.9	**10.4 ± 1.6**	10.1 ± 4.2
Bryozoans (B)	0	8 ± 3.5	12.6 ± 5.8	<0.1	8.5 ± 1.6	7.8 ± 1.9	**5.3 ± 1.2**	11.9 ± 3.8
CCA (B)	22.8 ± 3.9	22.3 ± 4.7	7.4 ± 1.2	17.5 ± 2.8	28.7 ± 5.5	10.8 ± 3.8	**20.2 ± 1.7**	36.8 ± 5.4
Corals (B)	0.2 ± 0.3	<0.1	0	0.2 ± 0.3	<0.1	0	**<0.1**	0.01 **±** 0.01
Fleshy algae (NB)	7.9 ± 4.2	3.1 ± 2.4	0	0.3 ± 0.1	3 ± 1.7	0.3 ± 0.2	**2.3 ± 0.8**	3.3 ± 2.3
Foraminifera (B)	0	<0.1	<0.1	0	<0.1	<0.1	**<0.1**	0
Mollusks (B)	0.5 ± 0.9	0.1 ± 0.2	0.1 ± 0.3	0.2	<0.1	0.5 ± 0.6	**0.2 ± 0.1**	0.3 ± 0.7
Non-serpulid Polychaeta (NB)	2 ± 2.8	<0.1	0.7 ± 1.2	1.4 ± 1.9	<0.1	0.2 ± 0.5	**0.7 ± 0.2**	<0.1
Serpulid Polychaeta (B)	0.2 ± 0.2	0.5 ± 1.3	2 ± 2.1	3.7 ± 11.8	<0.1	0.7 ± 1.1	**1.3 ± 0.8**	0.3 ± 0.2
Sponges—Calcarea (NB)	<0.1	<0.1	<0.1	0.1 ± 0.2	<0.1	0	**<0.1**	0.3 ± 0.2
Sponges—Demospongiae (NB)	1.8 ± 3.6	0.4 ± 1.2	<0.1	0	0.4 ± 0.7	0	**0.4 ± 0.2**	1.6 ± 1.1
Turfs (NB)	4.4 ± 1	2.5 ± 1.5	1 ± 0.4	21.6 ± 8	28.1 ± 3.2	17.8 ± 5.2	**14.1 ± 2.5**	21.1 ± 9
Zoanthids (NB)	0	0	0	0	<0.1	0	**<0.1**	<0.1
**Total abundance**	**52 ± 8**	**41 ± 7**	**41 ± 9**	**48 ± 8**	**77 ± 4**	**55 ± 8**	**52 ± 3**	**82 ± 5**

B = Builder organism; NB = Non-builder organism. Sites are indicated by letters.

The main groups of builder organisms were CCA and bryozoans; the non-builder organisms were ascidians, fleshy algae and turfs. All these groups of organisms showed a mean cover greater than 2% at all sites (Table 1).

After two years of colonization, the AA CAUs showed a percent cover of approximately 82 ± 5%. CCA and turfs were the most abundant groups, with a mean cover of 37% and 21%, respectively. Ascidians remained at more than 10% of mean cover, while bryozoans increased their cover from 5% (one year) to more than 10% (two years). In contrast, fleshy algae decreased their mean cover to 3%. Sponges, corals, mollusks and serpulid polychaetes remained less abundant, with mean cover below 2%. Non-serpulid polychaetes and zoanthids were rare, with less than 0.1% of mean cover. Foraminifers did not occur after two years of colonization (Table 1).

**Fig 6:**
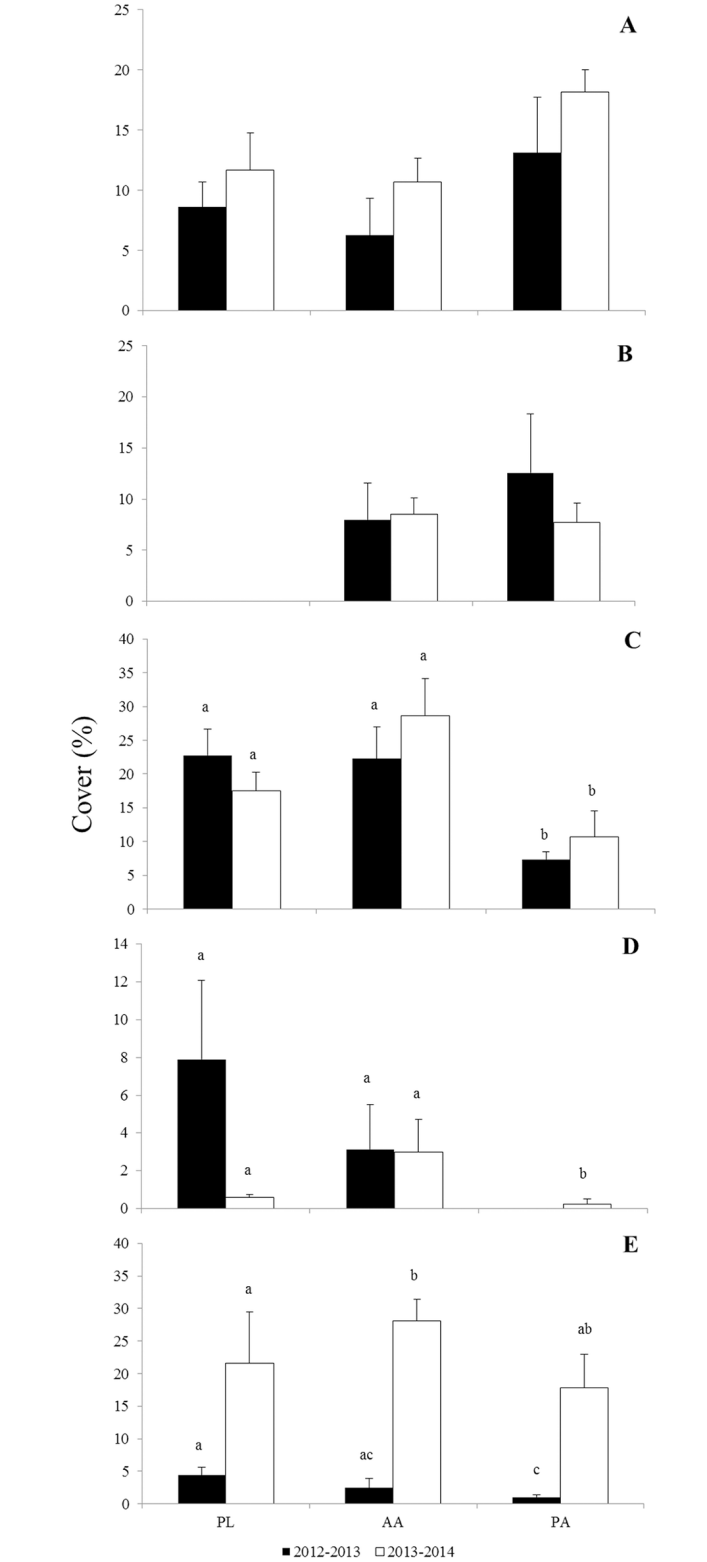
Cover (%) of main organisms at the sites (PL, AA and PA) after one year of colonization. A = Ascidians; B = Bryozoans; C = Crustose coralline algae; D = Fleshy algae; E = Turfs. Different letters above bars are used to indicate statistically differences (p< 0.05) of cover of organisms obtained through pair-wise tested after the PERMANOVA analysis.

Among builders, bryozoans did not occur in PL during 2012–2013, and they were rare during 2013–2014 (mean cover < 0.1%). In the sites where they occurred, AA and PA, no difference in the abundance was observed among sites (PERMANOVA; p = 0.7672; S3 Table; Fig 6) and years (PERMANOVA; p = 0.8944; S3 Table; Fig 6).

Crustose coralline algae percentage cover differed among sites, independent of the colonization year (PERMANOVA; p = 0.0004; S3 Table). No difference between the abundance at PL and AA was observed. These two sites presented higher CCA cover than that found at PA (Fig 6). The ascidian percentage cover did not differ among sites, nor in the different years (S3 Table, Fig 6).

The fleshy algae percentage cover differed among sites, regardless of the year when they were present (PERMANOVA; p = 0.0018; S3 Table). The highest abundance was observed at PL and AA, which did not differ from each other. PA presented the lowest cover among sites (Fig 6).

The turf percentage cover differed only between the years of colonization (PERMANOVA; p = 0.002; S3 Table). At both AA and PA, turf abundances were higher in 2014, fifteen times that of the previous year (Fig 6). At PL, no difference in turf covers was observed between the years. (Fig 6). After two years of colonization at AA, only the turf abundance increased (PERMANOVA; F = 4.6515; p = 0.0078). This was not the case with the other groups of organisms (S4 Table).

**Fig 7:**
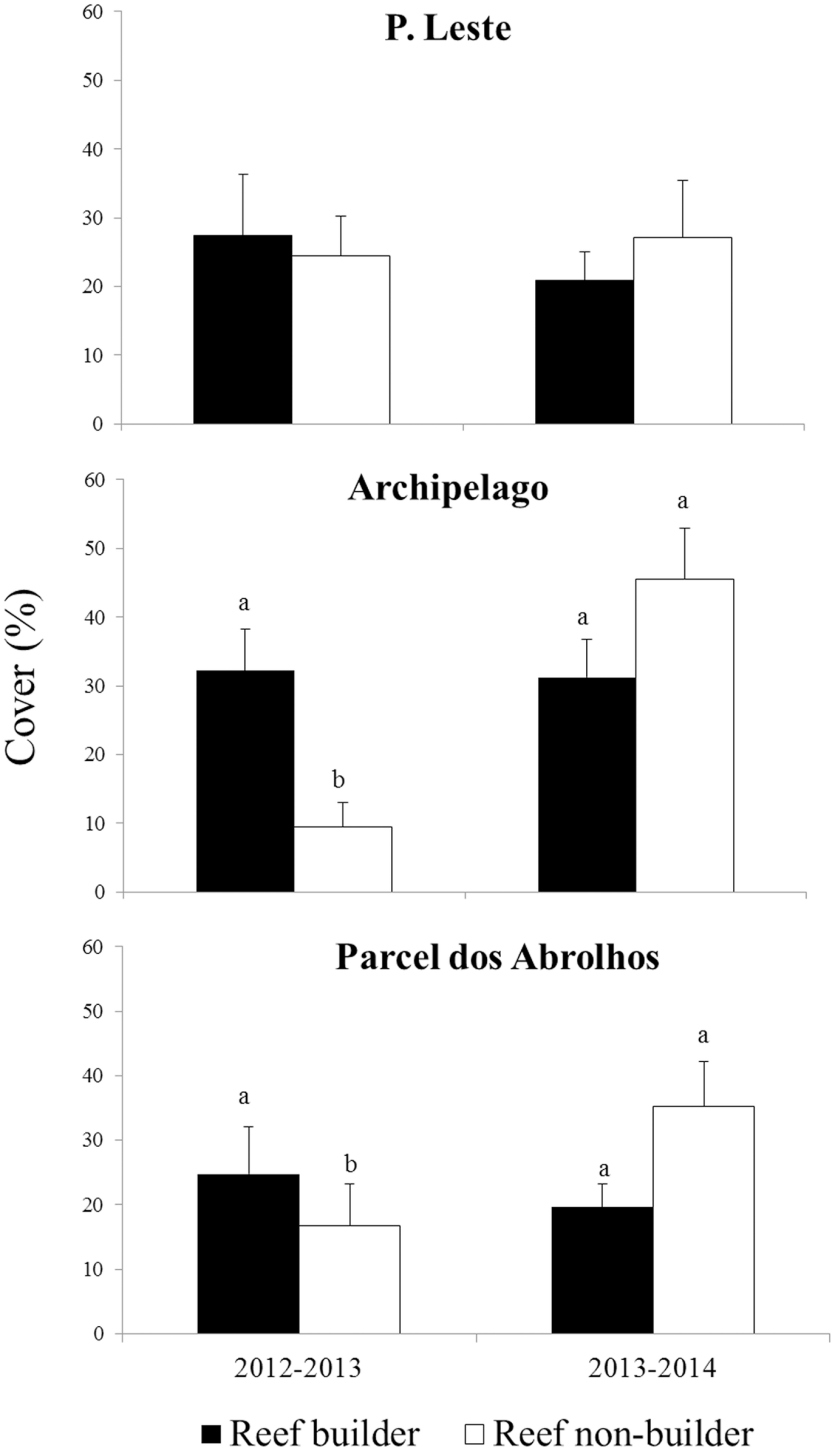
Abundance of reef-builder and non-builder organisms on shallow reefs (P. Leste, Archipelago and P. Abrolhos) after one year of colonization. Different letters above bars are used to indicate statistically differences (p< 0.05) of cover of organisms obtained through pair-wise tested after the PERMANOVA analysis.

The difference between builder and non-builder percentage cover varied according to the sites and the years (PERMANOVA; p = 0.0492, S3 Table). In PL, there was no difference between the cover of builder and non-builder organisms (Fig 7). However, at AA and PA in 2012–2013, the builder organisms were more abundant than the non-builders (Fig 7).

During 2012–2013, no differences in the abundance of builder organism groups or in that of non-builder groups were observed among the sites (S3 Table). During 2013–2014 period, the abundance of builder organisms did not differ among sites; however, the abundance of non-builder organisms was higher in AA, with a lower percentage cover in PL (S3 Table). The percentage cover of builder or non-builder organisms in PA did not differ from that at the other two sites (Fig 7).

After two years of colonization, the abundance of builder organisms in AA did not change in comparison with those after one year of colonization. However, the percentage cover of non-builder organisms was higher in the CAUs in 2014 (in both the one-year and the two-year experiment) than that in 2013, mainly due to turf abundance (S4 Table).

### Relationship between Environmental Factors and Abundance

A multivariate multiple regression (DISTLM) showed that the environmental variables contributing significantly to explain the differences in abundances among sites were light intensity (F = 3.1774; p = 0.0118) and temperature (F = 3.1291; p = 0.0013). Each of those factors explained 20% of the variability in reef community abundances among sites.

AA showed 22 days when the seawater temperature was above the summer average of 2012–2013 (> 26.8°C) and 27 days above the summer average of 2013–2014. However, higher temperature variation was detected in 2013–2014 based on a comparison of data from both summers. In the summer of 2012–2013, there were six consecutive days with the temperature above the average and none with temperature below the average (< 26.8°C). However, in the summer of 2013–2014, the temperatures were above 26.3°C during 18 consecutive days and below this average during 14 consecutive days. The turf cover increased at the CAUs in 2013–2014 and it was positively correlated with the increase in the number of consecutive days with temperature above and below 26.3°C (R = 0.957).

### Carbonate Production and Non-Calcareous Mass Produced in Shallow Reefs in Abrolhos

The mean carbonate production in shallow reefs of Abrolhos was 579 ± 98 g m^-2^ y^-1^. This carbonate production was higher in AA (728 ± 79 g m^-2^ y^-1^) in both time periods analyzed (PERMANOVA; p = 0.0476; S5 Table). The other two sites, PL and PA, both showed similar carbonate production. The mean non-calcareous mass produced on shallow reefs in Abrolhos was 109 ± 18 g m^-2^ y^-1^. This production was higher at both PL and AA regardless of year (PERMANOVA; p = 0.0126; S5 Table) (Table 2).

**Table 2:** Carbonate production (g m^-2^ y^-1^) and non-calcareous mass (g m^-2^ y^-1^) at sites PL, AA and PA after one year of colonization.

	Sites	Carbonate production (g m^-2^ y^-1^)	Non-calcareous mass (g m^-2^ y^-1^)
**2012–2013**	PL	437 ± 121	137 ± 26
	AA	745 ± 27	120 ± 6
	PA	455 ± 60	91 ± 19
**2013 2014**	PL	526 ± 5	127 ± 8
	AA	711 ± 120	108 ± 5
	PA	597 ± 78	71 ± 12

In the AA CAUs that remained two years in the field, the mean carbonate production at the end of this period (2012–2014) was 1377 ± 366 g m^-2^, higher than that for one year of colonization for the same site (PERMANOVA; F = 6.7885; p = 0.0378). In this period, 135 ± 20 g m^-2^ of non-calcareous mass was produced at the CAUs. This value did not differ from the values obtained after only one year of colonization (PERMANOVA; F = 2.1023; p = 0.2238).

## Discussion

After both one and two years of colonization, the most abundant groups were, in order of importance, CCA, turfs and ascidians. The bryozoans and fleshy algae were less abundant. Corals, sponges, mollusks, polychaetes, foraminifers and zoanthids were rare or did not occur on some plates.

Coral and CCA are considered the main builder organisms in Abrolhos [12,13]. However, on artificial substrates, coral cover was much lower than that of CCA and other colonial calcified organisms, such as bryozoans [50,36]. Mundy [51] and Field et al [52] observed a lower recruitment of different coral species after three and five months of colonization in artificial substrates. In contrast, in a study in the Central Pacific, no scleractinian coral recruitment was observed on similar colonization structures in non-degraded reefs [41]. Schumacher [53] suggested that a colonization period of one year is needed for coral recruitment on artificial substrates. Even after two years of colonization on CAUs in Abrolhos, corals were rare. This result is most likely related to the high cover of the other organisms, which decrease coral recruitment through competition. Coral recruitment and development can be inhibited by multiple competitive interactions among the species for the available substrate and resources [52, 54–56]. Furthermore, coral settlement can be inhibited or reduced by sedimentation and turf cover [32].

CCA were the most abundant organisms at all sites. CCA are pioneer colonizers of available hard substrates [57,58]. CCA are dominant on most Brazilian reefs and on Abrolhos reef, where their total cover ranged from: 4–36% [59], 2–15% [60], 3–40% [61], 10–20% [62] presenting a mean cover of 12% [26]. In other reefs from Bahia State (Brazil,) CCA cover was 30–40% [63], 30–49% [36] and in Atol das Rocas (Brazil): 36–60% [12] and 30–50% [64].

Bryozoans were rare or absent on the inner reef shelf (PL), where a higher amount of sediments were observed in the CAUs. Previous studies have also showed that bryozoans colonized only shaded substrates with low sedimentation rate [65–67]. Sedimentation and turbidity are the main factors that control bryozoan diversity and abundance [68]. Both factors interfere with the capability of the organism to obtain food and can cause smothering [69].

The amounts of sediments deposited on the colonized plates on the inner shelf reef were higher than on the outer reefs. Moreover, higher percentages of siliciclastic sediments, kaolinite (aluminum silicate) and quartz (silicon dioxide) were found on the inner reef shelf, while the carbonate minerals were most predominant on the outer reefs. Previous investigations showed no differences between the sedimentation rate on the inner reef shelf reefs and on the outer reefs on Abrolhos Shelf. However, the sediment composition varied apparently according to the site. While the outer reefs showed nearly 90% carbonate sediment, 40 to 70% of the sediment was siliciclastic on the inner shelf [70–72]. The kaolinite in PL is one of the possible components of clay-rich material and can be formed in tropical and temperate soils and in sedimentary basins [73]. The high abundance of kaolinite on the Abrolhos inner shelf confirms the influence of terrigenous sediments in this reef region. The most abundant mineral in the CAU sediments was Mg calcite in all sites, which may be due to the high abundance of crustose coralline algae in Abrolhos reefs.

The sizeable amount of ascidians colonizing the CAUs may seem peculiar at first, as these animals are usually present at lower densities or even absent from coral reef assessments [71]. In fact, ascidians are mostly cryptic animals that prefer shaded areas of crevices in the reef matrix [74,75]. Note also that most species found growing in the CAUs were didemnids, which produce a considerable amount of calcareous spicules (aragonite). Ascidians were abundant regardless of site and year, and their species composition may be considered diverse when compared with other taxa. Bryozoans, polychaetes and sponges are usually the main competitors for space with ascidians on the underside of recruitment plates, but some species are able to deter the recruitment process or simply grow over other organisms [76].

Regardless of the studied reef, turfs were the most abundant non-builder category of organisms. Turfs were dominant on the CAUs retrieved in the summer of 2014, and they were the only category of organisms that increased their cover after this period of colonization. The year of 2014 was the hottest since the beginning of temperature recordings by NOAA (National Oceanic and Atmospheric Administration). During this year, two atmospheric blocks—one in the Pacific and another in the Atlantic—prevented the passage of cold fronts in the southeastern region of Brazil during almost two months, between December of 2013 and February of 2014 [77]. During that summer, an extreme change in seawater temperature caused by a marine heat wave was detected. The seawater temperatures remained over 26.3°C during nine consecutive days of this period. It then decreased rapidly to below the average and remained at those temperatures for fourteen days; once again, it increased to above 26.3°C for 18 consecutive days. The number of consecutive days with temperature over or under the summer mean of 2013–2014 was positively correlated with turf cover increase, suggesting that temperature changes can contribute to the short-term phase shift observed in the shallow reefs of Abrolhos. An increase in turf cover has been observed at Abrolhos reef on the last decade [26]. The dominance of turfs, observed in several regions, can be explained by their strong ability to compete for space [78,79] due to their tolerance to physical and biotic stress [80–82] and their resistance to and fast recovery from disturbance [83,84]. On Abrolhos Bank reefs, turfs consist of an assemblage of different small seaweeds, filamentous cyanobacteria, microalgae and macroalgae in juvenile stages. These organisms show rapid growth due to their high vegetative propagation rate, which is a determining factor in terms of their capability to both compete for space and recover from disturbances [85].

IPCC [86] projections have indicated that oceans will be approximately 0.6°C to 2.0°C warmer by the end of the 21^st^ century. In addition, the global mean temperature anomaly is expected to be in the range of 0.3°C to 0.7°C for the 2016–2035 period relative to the 1986–2005 period. Considering that turf cover is increasing in the Abrolhos shallow reefs, these changes can accelerate the degradation process in reef communities.

The mean carbonate production at Abrolhos (579 g m^-2^ y^-1^) showed an intermediate value in comparison with other regions, higher than the estimated carbonate production in artificial structures in shallow reefs in the Caribbean, in Jamaica, 18–159 g m^-2^ y^-1^ [37] and in Mexico, 73–477 g m^-2^ y^-1^ [40], as well as in slightly degraded reefs in the Indo-Pacific, 468 g m^-2^ y^-1^ [87], and in French Polynesia, 584 g.m^-2^.y^-1^ [42]. However, the mean carbonate production at Abrolhos was less than values previously found in the Caribbean, in Tobago, 757 g m^-2^ y^-1^ [38] and in the Central Pacific, 700–1942 g m^-2^ y^-1^ [41]. A large range of carbonate production was found in Central and South Pacific from 40 to 2510 g m^-2^ y^-1^ which was the result of the variability of the physical and biological processes driving the structure and function of reef communities [55].

Calcimass was higher than non-calcareous mass, indicating that Abrolhos reefs are still in a positive calcium carbonate production balance and suggesting that production rates are higher than erosion rates. Perry et al. [3] evaluating gross and net carbonate production and erosion from 19 Caribbean coral reefs, showed that contemporary carbonate production rates are now substantially below historical (mid- to late-Holocene) values and suggested that a threshold of approximately 10% of living coral cover is necessary for a positive balance in carbonate production, as lower values can represent a threat to potential reef growth.

This study provides evidence that coral reefs on Abrolhos Bank are threatened by seawater temperature changes. Given that marine heat waves lead to an increase on turf cover on the shallow Abrolhos reefs, a decrease in reef-builder cover and shifts in long-term carbonate production are expected over the coming decades.

## Supporting Information

S1 Table Mineralogy of deposited sediments (%) on the CAU plates (mean ± standard error) in shallow reefs (PL, AA and PA) during 2013–2014. (DOCX)

S2 Table Taxa and species list of organisms colonizing CAUs, on Abrolhos Bank. (DOCX)

S3 Table Multivariate analysis results (PERMANOVA) after one year of colonization at all sites to test the effect of site, year and/or category (builder and non-builder group) on cover of main groups. Significant differences (p < 0.05) are highlighted in bold.
(DOCX)

S4 Table Multivariate results (PERMANOVA) after one and two years of colonization of AA to test the effect of colonization time period (1 or 2 years) and/or category (builder and non-builder group) on cover of main groups. Significant differences (p < 0.05) are highlighted in bold.
(DOCX)

S5 Table Multivariate analysis results (PERMANOVA) after one year of colonization at all sites to test the effect of site and year on carbonate production and non-calcareous mass. Significant differences (p < 0.05) are highlighted in bold.
(DOCX)
