# Supplementary material for: Carbonate Production by Benthic Communities on Shallow Coralgal Reefs of Abrolhos Bank, Brazil
Source: PLoS One. 2016 Apr 27;11(4):e0154417. doi: 10.1371/journal.pone.0154417 (PMC4847907; doi:10.1371/journal.pone.0154417)
Supplement: S1 Table — (DOCX) [file pone.0154417.s001.docx]

**Table S1. Mineralogy of deposited sediments (%) on the CAU plates (mean ± standard error) in shallow reefs (PL, AA and PA) during 2013-2014.**

| **Minerals** | **PL** | **AA** | **PA** |
| --- | --- | --- | --- |
| Calcite (%) | 5.4 ± 0.2 | 2.6 ± 1 | 6.4 ± 1 |
| Mg-calcite (%) | 43.6 ± 1.3 | 60.5 ± 1.5 | 52.6 ± 2.2 |
| Aragonite (%) | 35.5 ± 0.9 | 36.5 ± 1.1 | 40.6 ± 1.3 |
| Kaolinite (%) | 11.4 ± 1.3 | 0 | 0 |
| Quartz (%) | 5.1 ± 1.1 | 0.4 ± 0.06 | 0.4 ± 0.1 |
| **Mg-calcite/Aragonite Proportion** | **1.2 ± 0.04** | **1.7 ± 0.08** | **1.3 ± 0.1** |
