# Supplementary material for: Carbonate Production by Benthic Communities on Shallow Coralgal Reefs of Abrolhos Bank, Brazil
Source: PLoS One. 2016 Apr 27;11(4):e0154417. doi: 10.1371/journal.pone.0154417 (PMC4847907; doi:10.1371/journal.pone.0154417)
Supplement: S2 Table — (DOCX) [file pone.0154417.s002.docx]

**Table S2. Taxa and species list of organisms colonized on CAUS, in Abrolhos Bank.**

| **Ascidians** | *Stylopoma* sp.4 | **Polychaeta** |
| --- | --- | --- |
| *Ascidia* sp. | **Cnidarians** | *Branchiomma* *luctuosum* |
| *Botryllus* sp. | Zoantharia 1 | *Hydroides* spp. |
| *Corella* sp. | Zoantharia 2 | *Spirobranchus* *giganteus* |
| *Didemnum* *ligulum* | Agariciidae | **Sponges** |
| *Didemnum* *psammatodes* | Faviidae | Calcarea |
| *Didemnum* *speciosum* | Mussidae | *Chelonaplysilla erecta* |
| *Didemnum* *vanderhorsti* | Poritidae | *Clathria* sp*.* |
| *Diplosoma* *citrinum* | **Crustose calcareous algae** | Demospongiae 1 |
| *Diplosoma* *listerianum* | *Titanoderma prototypum* | Demospongiae 2 |
| *Diplosoma* sp. | *Hydrolithon reinboldii* | Demospongiae 3 |
| *Leptoclinides* cf. *brasiliensis* | *Porolithon onkodes* | *Halisarca* sp. |
| *Microcosmus* *exasperates* | *Peyssonnelia* spp. | **Turfs** |
| *Molgula* sp. | **Fleshy algae** | *Amphiroa anastomosans* |
| *Phallusia* *nigra* | *Canistrocarpus* sp. | *Amphiroa beauvoisii* |
| *Polycarpa* *spongiabilis* | *Dictyopteris* sp. | *Amphiroa fragilissima* |
| *Polysyncraton* sp. | *Dictyota* sp. | *Asparagopsis taxiformis* |
| *Pyura* cf. *munita* | *Lobophora* *variegata* | Cyanobacteria |
| *Styela* sp. n. | *Ochtodes* *secundiranea* | *Griffithsia* sp. |
| Styelidae | *Padina* sp. | *Jania adhaerens* |
| *Trididemnum* sp. | *Stypopodium* *zonale* | *Jania capillaceae* |
| **Bryozoans** | *Valonia* sp. | *Polysiphonia* sp. |
| *Bugula dentata* | *Wrangelia* sp. |  |
| *Celleporaria albirostris* | **Foraminifera** |  |
| Stenolaemata | *Homotrema rubra* |  |
| *Rhychozoon* sp. | **Mollusks** |  |
| *Stylopoma* sp.1 | Bivalvia |  |
| *Stylopoma* sp.2 | *Dendropoma* sp. |  |
| *Stylopoma* sp.3 | *Thylacodes* sp. |  |
