# Supplementary material for: Carbonate Production by Benthic Communities on Shallow Coralgal Reefs of Abrolhos Bank, Brazil
Source: PLoS One. 2016 Apr 27;11(4):e0154417. doi: 10.1371/journal.pone.0154417 (PMC4847907; doi:10.1371/journal.pone.0154417)
Supplement: S3 Table — Significant differences (p < 0.05) are highlighted in bold. (DOCX) [file pone.0154417.s003.docx]

**Table S3. Multivariate analysis results (PERMANOVA) after one year of colonization in all sites, to test the effect of site, year and/or category (builder and non- builder group) on cover of main groups.** Significant differences (p < 0.05) are highlighted in bold.

| Groups | Df | MS | F | p |
| --- | --- | --- | --- | --- |
| **Builders** |  |  |  |  |
| **Bryozoans** |  |  |  |  |
| Site | 1 | 9 | 0.0955 | 0.7672 |
| Year | 1 | 3 | 0.0286 | 0.8944 |
| Site x Year | 1 | 0.5 | 0.005 | 0.9786 |
| Residual | 44 | 105 |  |  |
| **CCA** |  |  |  |  |
| Site | 2 | 3550 | 4.6043 | **0.0004** |
| Year | 1 | 396 | 0.5132 | 0.6794 |
| Site x Year | 2 | 653 | 0.8464 | 0.504 |
| Residual | 66 | 771 |  |  |
| **Non-builders** |  |  |  |  |
| **Ascidians** |  |  |  |  |
| Site | 2 | 40 | 0.3816 | 0.696 |
| Year | 1 | 222 | 2.109 | 0.1496 |
| Site x Year | 2 | 30 | 0.2820 | 0.759 |
| Residual | 66 | 105 |  |  |
| **Fleshy algae** |  |  |  |  |
| Site | 2 | 367 | 6.9657 | **0.0018** |
| Year | 1 | 66 | 1.3259 | 0.2508 |
| Site x Year | 2 | 14 | 0.2884 | 0.756 |
| Residual | 66 | 49 |  |  |
| **Turfs** |  |  |  |  |
| Site | 2 | 147 | 1.679 | 0.191 |
| Year | 1 | 1016 | 11.616 | **0.002** |
| Site x Year | 2 | 264 | 3.0192 | 0.053 |
| Residual | 66 | 87 |  |  |
| **Builders x Non-builders** |  |  |  |  |
| Site | 2 | 1467 | 1.4847 | 0.189 |
| Year | 1 | 2583 | 2.6141 | 0.072 |
| Category | 1 | 4542 | 4.5972 | **0.0134** |
| Site x Year | 2 | 2221 | 2.2482 | 0.0618 |
| Site x Category | 2 | 227 | 0.22994 | 0.9588 |
| Year x Category | 1 | 4404 | 4.4578 | **0.0138** |
| Site x Year x Category | 2 | 2385 | 2.4142 | **0.0492** |
| Residual | 132 | 988 |  |  |
