# Supplementary material for: Carbonate Production by Benthic Communities on Shallow Coralgal Reefs of Abrolhos Bank, Brazil
Source: PLoS One. 2016 Apr 27;11(4):e0154417. doi: 10.1371/journal.pone.0154417 (PMC4847907; doi:10.1371/journal.pone.0154417)
Supplement: S4 Table — Significant differences (p < 0.05) are highlighted in bold. (DOCX) [file pone.0154417.s004.docx]

**Table S4**. **Multivariate analysis results (PERMANOVA) after one and two years of colonization, in AA, to test the effect of colonization time period (1 or 2 years) and/or category (builder and non- builder group) on cover of main groups.** Significant differences (p < 0.05) are highlighted in bold.

| Groups | Df | MS | F | p |
| --- | --- | --- | --- | --- |
| **Builders** |  |  |  |  |
| Bryozoans | 2 | 7 | 0,0905 | 0,923 |
| CCA | 2 | 375 | 1,0054 | 0,3728 |
| **Non-builders** |  |  |  |  |
| Ascidians | 2 | 235 | 0,2896 | 0,8276 |
| Fleshy algae | 2 | 5 | 0,136 | 0,8886 |
| Turfs | 2 | 4668 | 4,6515 | **0,0078** |
| **Builders x Non-builders** |  |  |  |  |
| Time period | 2 | 3122 | 3,8153 | **0,0042** |
| Category | 1 | 1826 | 2,2318 | 0,0856 |
| Time x Category | 2 | 3021 | 3,6917 | **0,0054** |
| Residual | 66 | 818 |  |  |
