# Supplementary material for: Carbonate Production by Benthic Communities on Shallow Coralgal Reefs of Abrolhos Bank, Brazil
Source: PLoS One. 2016 Apr 27;11(4):e0154417. doi: 10.1371/journal.pone.0154417 (PMC4847907; doi:10.1371/journal.pone.0154417)
Supplement: S5 Table — Significant differences (p < 0.05) are highlighted in bold. (DOCX) [file pone.0154417.s005.docx]

**Table S5. Multivariate analysis results (PERMANOVA) after one year of colonization in all sites, to test the effect of site and year on carbonate production and non-calcareous.** Significant differences (p < 0.05) are highlighted in bold.

|  | Df | MS | F | p |
| --- | --- | --- | --- | --- |
| **Calcimass** |  |  |  |  |
| Site | 2 | 677 | 3.2807 | **0.0476** |
| Year | 1 | 233 | 1.1296 | 0.3274 |
| Site x Year | 2 | 163 | 0.78994 | 0.504 |
| Residual | 12 | 206 |  |  |
| **Non-calcareous mass** |  |  |  |  |
| Site | 2 | 912 | 5.692 | **0.0126** |
| Year | 1 | 141 | 0.87974 | 0.368 |
| Site x Year | 2 | 45 | 0.28516 | 0.816 |
| Residual | 12 | 160 |  |  |
